# Dietary and lifestyle‐related factors behind delayed conception in females

**DOI:** 10.1002/fsn3.4274

**Published:** 2024-07-12

**Authors:** Maryam Khalid Rizvi, Roshina Rabail, Seemal Munir, Muhammad Waleed Rizvi, Gholamreza Abdi, Rana Muhammad Aadil

**Affiliations:** ^1^ National Institute of Food Science and Technology, University of Agriculture Faisalabad Pakistan; ^2^ Department of Applied Psychology Government College University Faisalabad Pakistan; ^3^ Department of Biotechnology, Persian Gulf Research Institute Persian Gulf University Bushehr Iran

**Keywords:** depression, folic acid, hypertension, infertility, iron, PCOS, physical activity, smoking, vitamin D

## Abstract

Delayed conceiving is a failure of a female to attain clinical pregnancy within a year without the use of contraception or any precautionary measure. The females of reproductive age (20–49 years) are the sufferers, that is, about 1.5 million or 10% of couples globally are facing this issue. Delayed conception can be caused by several reasons including diet, lifestyle, and genetic and clinical problems. Deficiencies or overconsumption of any nutrient may also cause delayed conception in females. Dietary deficits such as iron, iodine, zinc, folate, and vitamin D reduce the ovarian reserve. Heavy metals such as copper, manganese, lead, and cadmium also have an impact on fertility. Overconsumption of fast foods and nonhome‐made foods increases the likelihood of infertility by 2–3. Smoking, physical activity, age, polycystic ovary syndrome (PCOS), obesity, and depression have all been considered lifestyle and clinical factors. If women have a sedentary lifestyle, too much exercise or obesity both contribute to infertility. In clinical factors, type 1 diabetes, insulin resistance, and hypertension mainly cause infertility. Our focus in this review is on the studies probe that how these variables contributed to delayed conception and how it can be controlled to help combat infertility. Prevention from delayed conception involves consuming a healthy and balanced diet that contains essential nutrients engaging in physical activity and abstaining from smoking, PCOS, and medical issues.

## Graphical abstract

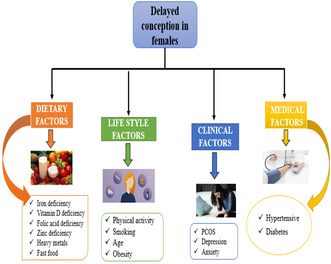

## 1 INTRODUCTION

Infertility is defined as the inability to conceive a child after 12 months or more of continuous, unprotected relationship, and it affects 15% of couples globally (Ashraf et al., 2024). According to the American Society of Reproductive Medicine (ASRM), infertility is the inability to conceive after 1 or more years of efforts at natural fertilization. Up to 20%–30% of viable females are affected by infertility, which is a serious problem in modern society. According to the World Health Organisation (WHO), this condition presently affects up to 80 million women worldwide, with developing nations accounting for 50% of all cases. Infertility affects women between the ages of 14 to 49 and is classified by the WHO as a disease of the reproductive system. According to ASRM, there are two kinds of infertility: primary and secondary. Primary infertility refers to a woman's inability to conceive, whereas secondary infertility is the term used for women who have given birth but are experiencing problems with their offspring (Manaf et al., 2023; Muratbayev et al., 2023; Ndeke et al., 2022; Silvestris et al., 2019). According to estimates, 8%–12% of couples globally are infertile, making infertility a universal health concern. Globally, estimates place the number of people living with infertility at 186 and 48 million, respectively (Wesselink et al., 2019). The longitudinal patterns of the primary and secondary infertility prevalence rate (PSIPR) per 100,000 people in all nations during the previous several decades. Compared to women, men had a lower PSIPR. Primary infertility rates in men and women showed a declining tendency over time, with rates in high‐income nations falling by −9.3 and −11.6, respectively. With rates of change of 40.9 and 19.0, respectively, South Asian women and males from the Middle East and North Africa have had the largest increases among other areas. With rates of change of −16.9 and −11.7, respectively, the prevalence of secondary infertility has been decreasing over time among women in high‐income nations, Central Asia, Central Europe, and Eastern Europe. Other regions have also seen increases, with South Asia, the Middle East, and North Africa seeing the largest increases among women (trends of 119.9 and 83.4, respectively) (Borumandnia et al., 2022).

Many nutritional deficits and lifestyle choices are risk factors that contribute to delayed conception. Iron deficiency is the most common dietary component that causes delayed conception. Iron deficiency anemia (IDA), which affects a large number of individuals globally, is mostly caused by a diet low in iron. A diet low in iron (Fe) affects the estrous cycle, which also lowers female fertility (Aboubakr et al., 2023; Liaqat et al., 2023; Tonai et al., 2020). It has been demonstrated that folic acid has an effect throughout the whole pregnancy. Low body folic acid levels lower the incidence of embryonic neural tube defects, which has decreased the occurrence of abortions. Additionally, ovarian processes may be impacted by folic acid (Liao et al., 2022). Heavy metals also associated with delayed conception in females such as low levels of cadmium and lead in blood may cause infertility in females. And also affects the ovarian reserve in females (Lee, Min, & Min, 2020). When discussing the lifestyle and clinical variables that contribute to delayed conception, the first and most significant is the duration of obesity, which influences the degree of ovarian dysfunction. Prolonged obesity impairs ovulation and folliculogenesis, increasing reproductive issues (Legro et al., 2022).

Worldwide, there is a high prevalence of reproductive and metabolic problems. They are all caused by the same things: obesity, an excess of nutrients, and a decrease in energy consumption. Obesity has detrimental effects on the reproductive system that vary depending on the quantity and location of body fat. Diabetes mellitus, irregular menstruation, infertility, miscarriage, poor pregnancy outcomes, and decreased fetal well‐being are all associated with obesity. Reproductive and metabolic problems place a heavy social, medical, and financial cost on people and society. Numerous reproductive health issues are linked to diabetes, including subfertility, poor pregnancy outcomes, irregular menstrual cycles, delayed puberty and menarche, and perhaps early menopause. The use of supplements would be very beneficial for reproductive organs (El‐Dawy et al., 2023; Farooq et al., 2022; Khan et al., 2022; Shalaby et al., 2022).

Although there has been a link between in vitro fertilization and a higher risk of hypertensive disorders during pregnancy, the relationship between risk and IVF treatment parameters is unclear. For women who have had IVF, the risk of hypertensive disorders of pregnancy is elevated in both fresh donor oocyte and frozen embryo transfer‐conceived pregnancies. Controlling for body mass index (BMI) and infertility diagnoses, excluding women with pregestational diabetes or severe hypertension, and adjusting for these factors did not significantly alter the findings (Luke et al., 2020). Women who choose to live sedentary lives throughout their reproductive years instead of participating in regular physical activity, regardless of intensity, have changed hormone patterns that affect their physical well‐being. The ovarian hormone pool might decrease with age and inactivity. However, excessive physical activity also has an impact on fertility, especially in women with low ovarian reserves (Zubair et al., 2021).

Hazardous heavy metals including arsenic, cadmium, mercury, lead, zinc, and copper can harm the ability of most species to reproduce. Natural processes like rock weathering and volcanic eruptions as well as human activity like industrial discharge, mineral mining, and automotive exhaust all release heavy metals into the environment. Heavy metals affect several reproductive processes in both males and females, including sperm motility, viability, spermatogenesis, hormonal imbalance, follicular atresia, and delayed oocyte maturation, among others. As a result, these can play a significant role in reproductive toxicology (Ali et al., 2024; Bhardwaj et al., 2021; Samy et al., 2022). Infertility is a significant issue in the current society that impacts a large number of couples globally. Infertility has been causally connected to heavy metals and a variety of other causes. The number of infertile people has risen in the past few years. There are several potential environmental, occupational, and genetic contributing factors. Couples who are exposed to lead, cadmium, or copper have infertility due to a variety of factors, including modified sperm motility parameters, reduced semen quality, or effects on the egg. The beginning of infertility may also be influenced by exposure to physical phenomena such as heat, ionizing radiation, and respiratory pollutants like lead and lead paint, as well as stress, mental health issues, anesthetic gases, mercury, and cytotoxic medications (Lauriola et al., n.d.).

It is very difficult to identify delayed conception in females. For screening out the cases of delayed conception, different methods have been used. Some of these methods including deep scientific insights into the experiences of delayed conception, coping techniques, medical support, and other help sought are obtained. Furthermore, data on socio‐demographic characteristics, reproductive goals, reproductive quality of life, general medical history, obstetric, gynecological, sexual histories, substance use history, and mental health status are collected. This information is very helpful for the identification and screening of infertility in females (Adhikary et al., 2022).

Therefore, this study aims to identify the possible risk factors behind the occurrence of delayed conception cases in females. The objectives are to highlight the possible direct or indirect interrelationship between dietary and lifestyle factors or to identify the coexistence of unhealthy dietary consumption and lifestyle patterns along with delayed conception. This review is therefore written to bring forward the previous scientific studies that can help in identification of conception delay. For this purpose, research articles of the last 5 years were downloaded using Google Scholar, Scopus, etc., using the following keywords: “delayed conception” OR “delayed pregnancy” OR “primary infertility” OR “secondary infertility” AND “diet” OR “lifestyle” OR “nutrition**”** OR “clinical issues” etc.

## 2 RISK FACTORS ASSOCIATED WITH DELAYED CONCEPTION

The risk factors associated with this delayed conceiving include several dietary deficiencies and lifestyle patterns.

### 2.1 Diet‐related risk factors

Many scientific researchers have connected the possibilities of delayed conception with certain micronutrient deficiencies. Among those dietary factors, the most prominent is an iron (Fe) deficiency.

#### 2.1.1 Iron deficiency

Fe is required for a variety of biological processes, including adenosine triphosphate (ATP) production and cell growth. IDA is a prevalent condition affecting people all over the world, especially females at reproductive age. It is caused by a diet low in Fe. About 50% of women with IDA experience amenorrhea. In low‐Fe diet mice, the diestrus stage (the last, or luteal, stage of the cycle, starts around the 14th day of the estrous cycle, or whenever estrus ends) of the estrous cycle inhibited follicle development and decreased fertility. Compared to mice fed a normal diet, the ovary of the animals on the low‐Fe diet had less ATP, lower levels of follicle development markers (Fshr, Cyp19a1, and Ccnd2), and lower levels of estradiol‐17 expression. After being fed a regular diet with just adequate Fe for an additional 3 weeks, the effects of Fe deficiency on the estrous cycle and infertility were recovered in the mice on the low‐Fe diet. Therefore, Fe limitation inhibits ovarian function, particularly the transition from secondary to antral follicles and infertility. These effects can be completely reversed by adding Fe supplements to a typical diet (Tonai et al., 2020).

The follicular fluid is the ovum's surrounding environment, and its content can be both beneficial and harmful. Because Fe plays an important role in providing oxygen to the oocyte, its presence in the follicular fluid reduces the chances of Fe playing a crucial function in giving the oocyte oxygen, thus having it in follicular fluid lowers the likelihood of conception as shown in the PCOS group, and raises the rate of in vitro fertilization (IVF) failure. If pregnant, as seen in the PCOS group, this increases the percentage of in vitro fertilization (IVF) failure (Kadhum & Al‐Shammaree, 2021). In another study, the relationship between Fe deficiency and reproductive function was examined using mice fed a low‐Fe diet, even though amenorrhea affects 50% of women with IDA. During diestrus, the low‐Fe diet mice's estrous cycle was set off, which reduced follicle development and fertility. Furthermore, exogenous pregnant mare serum gonadotropin (PMSG) was fed to low‐Fe diet animals, however, follicular development stopped at the secondary follicle stage and no preovulatory follicles were seen. Compared to animals fed a standard diet, low‐Fe diet mice had lower levels of ATP in their ovary and lower expression of estradiol‐17 and follicle development markers. The effects of Fe deficiency on the estrous cycle and sterility were entirely reversed by feeding the low‐Fe diet mice a regular diet with enough Fe for an additional 3 weeks. Hence, Fe deficiency impairs infertility as well as ovarian function, especially follicular development from secondary to antral follicles (Tonai et al., 2020).

#### 2.1.2 Vitamin D deficiency

**FIGURE 1:**
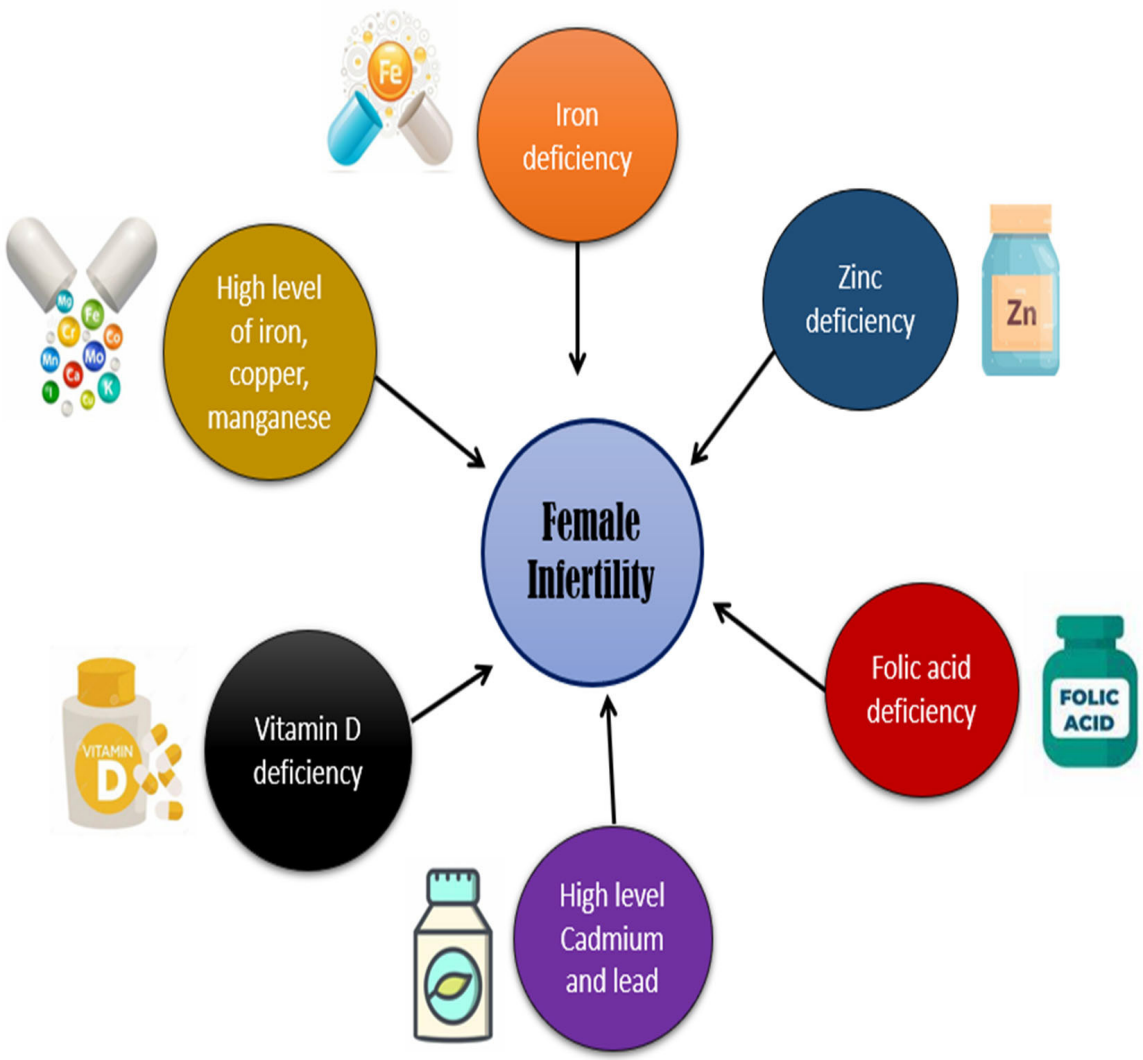
Nutritional factors that lead to delayed conceiving.

One of the steroid hormones is vitamin D. The skin produces around 80% of its vitamin D when exposed to sunlight. Additionally, a small amount of vitamin D is obtained via food and/or dietary supplements. For its part in phosphor‐calcic metabolism and bone mineralization, vitamin D is well recognized. In both men and women, vitamin D has an impact on reproduction. Both male and female reproductive organs express vitamin D receptors and the enzymes that regulate vitamin D metabolism, which may indicate that vitamin D may have an impact on sperm quality, fertility, and conception rate. The role of vitamin D in both male and female fertility is described in this article (Sebbar & Choukri, 2023). Figure 1 shows the nutritional factors that lead to delayed conceiving.

In many areas of medicine, particularly reproductive medicine, vitamin D has arisen as a major topic and a research priority. Studies in reproductive medicine have found a link between women's vitamin D levels and ovarian reserve. The greatest significant source of vitamin D in humans is exposure to sunlight. A risk factor for vitamin D insufficiency is dressing in protection all the time. Participants in the study got transvaginal ultrasounds on days 2–5 of the cycle to determine the antral follicle count, as well as blood tests to quantify vitamin D content. An expert in reproductive medicine and a consultant dermatologist evaluated each trial participant to determine their skin type according to the Fitzpatrick classification. Each research participant's dressing was also documented, including the amount of exposed flesh that was not covered by a covering fabric. A concealing dressing code raises the risk of vitamin D insufficiency by preventing the skin from getting adequate sunlight. As a result, a significant vitamin D shortage might be a primary contributor to diminished ovarian reserve (Arefi et al., 2018).

In another retrospective analysis, 274 PCOS‐affected infertile women and 111 fertile women were included. Age, BMI, and the measurement of insulin resistance using a homeostasis model were used to match infertile and fertile groups. The women's anthropometric, clinical, and laboratory features were noted. Serum vitamin D levels were determined by measuring the concentration of serum 25(OH)D3. Serum vitamin D levels were lower in PCOS‐affected infertile women than in fertile ones. The presence of insulin resistance or obesity further reduces vitamin D levels. Thus, PCOS pathogenesis of reproductive problems may be affected by lower vitamin D levels (Kokanall et al., 2019). Comparable research reveals that low vitamin D levels in PCOS harm fertility. A study included 146 infertile women who were separated into two groups based on the patterns of their ovarian reserve: normal (NOR) and high (HI). In addition, the individuals were divided into two groups based on their vitamin D levels: (i) Group A, 10 ng/mL, and (ii) Group B, 10–20 ng/mL. Estradiol (E2), follicle‐stimulating hormone (FSH), luteinizing hormone (LH), total testosterone (TT), 17‐hydroxyprogesterone (OHP), dehydroepiandrosterone sulfate (DHEA‐S), and anti‐Mullerian hormone (AMH) levels were measured in the samples. The anthropometric characteristics of Group A and Group B differ markedly from one another. Both Group A's and Group B's female participants had similar AMH concentrations. Only the NOR group demonstrated a substantial but moderately unfavorable connection between anthropometric measurements and 25(OH)D levels. In the NOR or the PCOS group, there was no correlation between AMH levels and 25(OH)D levels. A multivariate regression analysis revealed that 25(OH)D did not influence the other research parameters. To distinguish between PCOS and NOR patterns, only AMH showed significant results (*p* .001). Concluded that in women with and without PCOS, there was no change in serum 25(OH)D and AMH levels, according to our research. No relationship between 25(OH)D and AMH in the PCOS group or controls could be proven (Arslan et al., 2019).

Moreover, women from the prospective population‐based Northern Finland Birth Cohort 1966 who were 31 years old were included in this study. With a variety of confounders, serum 25(OH)D concentrations were compared between women who had or had not previously had infertility tests or treatments (infertility group, *n* = 375, reference group, *n* = 2051) and those who had delayed becoming pregnant by more than 12 months (decreased fecundability group, *n* = 338). Additionally, differences in 25(OH)D concentrations were seen among reproductive outcomes. Compared to the reference group, women with a history of infertility had a lower mean 25(OH)D concentration and a higher frequency of 25(OH)D < 30 nmol/L. Furthermore, there was a higher frequency of 25(OH)D > 75 nmol/L in the reference group. Women who have experienced several miscarriages had decreased mean 25(OH)D concentrations. Following modifications, a lower 25(OH)D concentration was linked to both a history of infertility and impaired fecundability (Lumme et al., 2023).

Similarly, this cross‐sectional study involved 325 infertile women who were referred to the Yas hospital's IVF Unit between July and December 2017. Together with the blood levels of vitamin D, and AMH, and an ultrasonic examination of AFC, the participant demographics were also noted. The age of the participant is between 18 and 45 years old and their BMI is 25.11 ± 4.41 kg/m^2^. Even when baseline factors were taken into account, regression analysis did not reveal a relationship between AMH and vitamin D levels. Even after controlling for baseline factors, there was no correlation seen between AMH and vitamin D levels in 120 individuals with an AFC < 6 and 164 with a ≥6. With a cut‐off level of 20 ng/mL, 120 patients had an AFC < 6 and 164 > 6, meaning that 58.7% of them were vitamin D deficient. Even when baseline factors were taken into account, regression analysis did not reveal a relationship between AMH and vitamin D levels. There were 120 patients with an AFC < 6 and 164 with an AFC ≥ 6, and there was no significant difference between the groups with normal or inadequate vitamin D levels. Even after controlling for baseline factors, the current cross‐sectional investigation found no evidence of a significant correlation between blood vitamin D levels and AMH or AFC in infertile women (Alavi et al., 2020).

In another study, 284 individuals with infertility issues had their files reviewed for this retrospective research. Group I included patients whose vitamin D levels were less than 20 ng/mL, while Group II comprised all other patients. Those with vitamin D levels more than or equal to 20 ng/mL were compared as a group. The groups' vitamin D levels were assessed based on age, and throughout the summer, seasonal analysis was conducted. The level of estradiol (E2) was found to be *p* < .013. Furthermore, no statistically significant relationship was seen between vitamin D and antral follicle number (AFC), follicle‐stimulating hormone (FSH), or AMH. It was shown that the vitamin D levels of female patients who came to our clinic because they were infertile did not significantly correlate with the markers of ovarian reserve, particularly AMH, FSH, and AFC. New data for therapeutic applications are needed from multicenter and prospective research on vitamin D, which is crucial for infertility in both males and females (Özçelik & Özdemir, 2024).

#### 2.1.3 Zinc deficiency

Several conserved systems that control the development of female germ cells, ovulation, and pregnancy depend heavily on zinc. A sufficient intracellular concentration of zinc in the oocyte during follicle growth maintains meiosis arrested at prophase I until the germ cell is ready to go through maturation. Due to the disruption of maturation and lower oocyte quality caused by dietary zinc shortage or chelation, an adequate supply of zinc is required for the oocyte to develop into a fertilization‐competent egg. The zinc spark, a rapid release of zinc that occurs after sperm and egg fusion to start the acrosomes reaction, promotes egg activation in addition to increasing zona pellucida hardness and decreasing sperm motility to prevent polysemy. Zinc availability is essential for the preimplantation embryo's symmetric division, proliferation, and differentiation, both throughout the development of the oocyte and after fertilization. Also, in pregnant women who struggle with zinc shortage, the development of the neural tube, fetal limb growth, and placental contribution are all hampered. For women of reproductive age who are at risk of not consuming enough zinc, supplementation with zinc should be considered (Garner et al., 2021).

In another study, 29,400 people who completed the 2013–2018 National Health and Nutrition Examination Surveys were assessed for this national survey. The subjects were split into two groups: low zinc and high zinc (<8 and ≥8 mg/day, respectively) based on recommended dietary requirements for zinc consumption; also, groups were formed based on BMI: low BMI and high BMI. Infertility risk was higher among those who were exposed to pesticides at home. A zinc‐rich diet may lessen the risk of pesticide‐induced infertility since the frequency of infertility varied between the low‐ and high‐zinc groups, suggesting a relationship between household zinc consumption and pesticide exposure. The low‐BMI group showed a different association between pesticide exposure and infertility risk than the high‐BMI group, indicating that high BMI may exacerbate the risk of infertility put on by pesticide exposure. These new data show that obesity and poor‐zinc diets have a synergistic effect on the likelihood of pesticide‐induced infertility, respectively, and that obese people may be more vulnerable to infertility caused by household pesticide exposure (Huang et al., 2023).

#### 2.1.4 Folic acid deficiency

Folic acid belongs to the family of vitamin B and controls the one‐carbon cycle and methylation. Folic acid has been proven to have an impact during the whole pregnancy, and supplementation has been demonstrated to significantly reduce the frequency of fetal neural tube abnormalities and lower plasma homocysteic acid levels, which has led to a drop in the prevalence of abortion. Folic acid is also thought to affect ovarian processes. Folic acid may therefore help with diminished ovarian reserve. A randomized, open‐label, single‐center, placebo‐controlled clinical study is developed. A total of 140 women with diminished ovarian reserve (DOR) between the ages of 30 and 35 are the target population. Each individual will get a daily pill with the same appearance for 6 months after being randomly assigned to either the folic acid or placebo groups. The number of antral follicles is the main result; ovarian reserve indicators, ovarian low‐dose stimulation responses, and safety are the secondary results (Liao et al., 2022).

In another study, 120 women between the ages of 18 and 39 were in the reproductive cycle. They were divided into three groups: 40 adult females without PCOS were examined in Group 1, 40 adult females with PCOS who were not treated for 3 months with 5 mg/day of folic acid were studied in Group 2, and 40 females with PCOS who were not treated for 3 months with 5 mg/day of folic acid were included in Group 3. Malondialdehyde (MDA), a result of lipid peroxidation, was shown to be considerably reduced in PCOS women who were given folic acid at a dose of 5 mg per day (Al‐mosawi, 2021).

#### 2.1.5 Heavy metals toxicity

Exposure to heavy metals is a risk factor for infertility that can harm both male and female reproductive systems. However, little research has been done on the relationship between exposure to heavy metals and female infertility. Utilizing information from the National Health and Nutrition Examination Survey's three cycles (2013–2018), a cross‐sectional research was conducted. Positive answers to questionnaire item rhq074 were used to assess female infertility. Using inductively coupled plasma–mass spectrometry, the amounts of cadmium (Cd), lead (Pb), mercury (Hg), and arsenic (As) in blood or urine were measured. With weighted logistic regression, the relationship between heavy metal exposure and female infertility was examined. Elevations in urine As were linked to a greater risk of infertility in females, and urinary As was shown to be strongly connected with infertility. Urinary Cd and infertility were somewhat associated. Blood/urine lead levels were linked to infertility in older and obese/overweight women (Lauriola et al., n.d.).

In this study, a total of 91 patients with primary infertility, 169 pregnant women, 150 healthy controls, 75 women who had miscarried, and 150 pregnant participants were enrolled. Metal concentrations in the serum were measured using inductively coupled plasma mass spectrometry (ICP‐MS). Copper (Cu) and manganese (Mn) serum levels are 40% and 16% higher in pregnant women than in nonpregnant women, respectively. Infertile and miscarried women had serum Cu levels that were, respectively, 35% and 30% lower than those of pregnant women. There was no significant difference in the serum Fe levels between the pregnant women and the control group. In comparison to pregnant women, the serum iron levels of miscarriage women were 13% higher. The results of multiple regression analysis showed that blood copper levels were substantially related to both pregnancy and problems with female reproductive health. After taking into account the serum levels for Mn, Fe, BMI, and, age, and, the latter significantly improved. Twenty‐three percent of the variation in reproductive status could be explained by a model that included anthropometric measures and serum Cu, Fe, and Mn. The absence of an increase in metal levels during pregnancy in cases of miscarriage and infertility is said to suggest that a metal deficiency contributes to poor pregnancy and reproductive function overall (Skalnaya et al., 2019).

This study was investigated to check the relationship of lead and cadmium in the blood of infertile and pregnant US women and self‐reported infertility women. Women aged 20–39 who took part in the 2013–2014 and 2015–2016 waves of the National Health and Nutrition Examination Surveys had their blood lead, blood cadmium, and infertility levels examined (*n* = 124, “pregnant” and *n* = 42, “infertile”; *n* = 82). Infertility and pregnancy status were evaluated using a self‐reported questionnaire, and blood lead and cadmium levels were determined using inductively coupled plasma mass spectrometry. After controlling for confounding factors, low blood levels of lead and cadmium were found to be positively associated with self‐reported infertility. Our results imply that even low blood levels of cadmium and lead may be harmful to female fertility, however, they still need to be confirmed (Lee, Min, & Min, 2020).

#### 2.1.6 Fast/junk food consumption

Fast food, ready‐to‐eat foods, and frozen foods are examples of nonhome‐prepared meals, which have been linked to self‐reported infertility in American women. There was a positive correlation between the frequency of nonhome‐prepared meal use and self‐reported infertility. Women who ate fast food had risks of infertility that were two to three times higher than those who did not. The kind and frequency of nonhome‐prepared meals may play a role in infertility. Data were collected from infertile women 20–49 years old who took part in the National Health and Nutrition Examination Surveys in 2013–2014 and 2015–2016 were examined. A self‐reported questionnaire was used to gather dietary information, including the quantity and varieties of nonhome‐prepared meals ingested, and the questions related to fertility status were added, for example, “Have you ever tried to get pregnant for at least a year without becoming pregnant?” was used to examine infertile status. After controlling for confounding factors, the frequency of nonhome‐prepared meal use was shown to be positively linked with self‐reported infertility. Women who ate fast food had risks of infertility two to three times higher than those who did not. Many factors cause infertility when you frequently consume nonhome‐made food (Lee, Min, Kim, & Min, 2020).

In another study, infertile women exercised regularly, whereas 44.9% of fertile women had a healthy lifestyle. Females ate three times a day. Healthy food was consumed once a week, whereas 35% of fast food was consumed around twice a week. Those who consume unhealthy diets and fast food have more chances of being infertile as compared to those who consume a healthy diet. Fertility is favorably connected with optimal physical activity and a BMI in the normal range (Table 1). Increasing consumption of fish, fruits, and vegetables has a good influence on fertility (Alam et al., 2021).

**TABLE 1:** Dietary factors that cause delayed conception.

Factors	Subjects	Study design	Objective	Methodology	Result	References
Fe deficiency	3‐week female mice	Random control trails	For investigations, low‐Fe status influences follicular development.	Providing Fe supplements with a normal diet in low‐Fe diet in mice for 3 weeks.	Fe supplements help increase ovary function and follicular development.	Tonai et al. 2020)
Fe	99 females divided into 3 groups (PCOS, control group, and low AMH)	Case–control prospective study	To assess the levels of Fe and ceruloplasmin ferroxidase in the follicular fluid (FF) and their relationships to the success of IVF	Asses the Fe status in 3–4 groups of females (ages between 22 and 38 years) in a case–control study. For checking, Fe influences fertility in women, especially infertile women undergoing IVF/ICSI.	Fluid present in the surrounding ovum. Fluid decreased the chances of pregnancy in the PCOS group and raises the failure rate of the IVF procedure.	Kadhum & Al‐Shammaree (2021)
Folic acid + PCOS	120 women at reproductive age (18–39)	Cross‐section study was carried	Folic acid effects on lipid peroxidation in women with PCOS	Women were divided into three groups and provided orally 5 mg of folic acid per day	Folic acid supplementation can considerably lower the rate of lipid peroxidation.	Al‐mosawi (2021)
Vitamin D deficiency + PCOS	111 fertile women with PCOS and 274 infertile women	Retrospective case–control study	Evaluated that vitamin D contributes to infertility	PCOS females were referred to the Reproductive Endocrinology and Infertility Department in Ankara, Turkey, due to PCOS‐related symptoms over 2 years A study was conducted on those who take vitamin D, contraceptives, and drugs	Lower vitamin D levels may contribute to fertility issues in PCOS. Except for serum 25(OH)D3 levels, no significant variations in anthropometric clinical laboratory characteristics were found across groups.	Kokanall et al. (2019)
Vitamin D	284 infertile women	A retrospective cohort study	To evaluate the relationship between vitamin D level, and AMH and other ovarian reserve parameters impact infertility.	Infertile women were divided into two groups Group I with vitamin D lower than 20 ng/mL and Group II with above 20 ng/mL Their AFC, AMH, and FSH levels were measured	No significant relationship between ovarian reserve and delayed conception.	Özçelik & Özdemir (2024)
Obesity + older age + ovarian disorder + iodine deficiency	330 infertile overweight women.	Cross‐sectional study	To study the epidemiological aspects of infertility and associated risk factors in infertile women.	From April 2015 to March 2017, infertile women were directed to two infertility treatment facilities at Imam Khomeini Hospital and Mother Center in Sari, Iran. For examination of which factors cause infertility in females in the region of Iran.	Infertile women were older, less educated, and overweight in the north of Iran. Their chances of acquiring ovarian diseases are higher. Thyroid illness is more prevalent in iodine‐deficient regions.	Marzieh et al. (2021)
Copper, Fe, and manganese	Women 485	Cross‐section study	Evaluate how high and low level of metals leads to infertility and reproductive problems	ICP‐MS was used to assess the metal level in the body. After an overnight fast, blood samples were drawn from the cubital vein using “Vacuette” tubes The serum was then centrifuged at 1600 ***g*** for 10 min to obtain blood for analysis.	High levels of metals cause infertility, miscarriages, poor reproductive, and pregnancy function	Skalnaya et al. (2019)
Cadmium and lead levels	10,251 women (20–39 years)	Cross‐section study	Female infertility linked to elevated blood lead and cadmium levels	Compares metal levels in infertile and pregnant women to investigate the relationship between self‐reported infertility and blood lead and cadmium levels in US women	There was a significant correlation between low blood lead and cadmium levels and infertility	Lee, Min, & Min (2020)
Vitamin D	149 infertilities females (18–35 years)	Prospective cross‐sectional study	Is there a link between PCOS and vitamin D deficiency in infertile women?	To perform this survey, female participants were separated into two groups based on their ovarian reserve Group A has a severe vitamin D deficiency. Whereas Group B has a mild vitamin D deficiency In HITIT University Hospital in Orum, Turkey.	In the PCOS group and control group, no relationship between 25(OH)D and AMH could be seen	Arslan et al. (2019)
Vitamin D	305 infertile women	Prospective observational study	How does vitamin D status affect the ovarian reserve?	This study was conducted in the Unit of Yas Hospital; their serum vitamin D level and ultrasonic examination of AFC and AMH were recorded	No association between AMH, AFC, and vitamin D with fertility in females	Alavi et al. (2020)
Fast food	Infertile women aged 20–49 years	Cross‐section study	Does fast food cause infertility?	Data were collected by using a questionnaire that included dietary informational Questions related to infertility	Frequently eating nonhome‐made meals caused infertility	Lee, Min, Kim, & Min (2020)

### 2.2 Lifestyle and clinical factors

#### 2.2.1 Physical activity

Idiopathic infertility (a condition where a clinical evaluation reveals no abnormal findings) is associated with physical inactivity in males and sedentary behavior in women. Body fat buildup has been related to infertility in both men and women, but the fat‐free mass has only been linked to infertility in women. Physical inactivity and sedentary behavior are two independent risk factors for infertility, according to this case–controlled study. Lifestyle changes in body composition should be studied further about the biological mechanisms implicated in idiopathic infertility. The effects of a sedentary lifestyle and physical activity on fertility do not always remain constant. An elevated body mass index is linked to infertility. An observational multicentric case–control research was carried out. Four fertility centers recorded 159 infertile people (79 men and 80 women) and 143 fertile people (72 men and 71 women). Self‐administered questionnaires on dietary consumption, physical activity, sedentary behavior, and sociodemographic and lifestyle factors were filled out by participants. Body composition was estimated using bioelectrical impedance analysis and anthropometric measurements. Utilizing multivariable logistic regression, the relationship between fertility sedentary behavior and PA level was examined. Studies show that a sedentary lifestyle is independently linked to the cause of delayed conception. Additionally, while treating infertility, lifestyle variables should be improved. These data show that to enhance pregnancy rates, lifestyle‐supportive care should be promoted and offered throughout fertility treatment (Foucaut et al., 2019).

In another study, a case–control study including 320 married participants was carried out in the Gaza Strip. Between 2016 and 2018, 160 infertile couples were selected from five fertility centers' registries and paired up residentially with 160 fertile couples. Five lists were categorized according to domicile, and cases were chosen using a systematic stratified selection from each list at a proportionate rate. Data were gathered using a self‐administered survey that was expanded by the short form of the international physical activity questionnaire, and they were analyzed using the SPSS program version 22 with the help of cross‐tabulation, independent T‐test, and binary logistic regression. Low physical activity levels were linked to a 3.1% increased incidence of infertility in the first trimester. Reproductive issues were 2.3 times more prevalent in women who sat sedentary for more than 300 min per day than in those who engaged in physical activity. The reproductive status of women in the Gaza Strip is at risk due to low physical activity levels and sedentary lifestyles. This may point to the necessity of adopting and formalizing appropriate physical activity education and awareness protocols in the national reproductive health recommendations as well as strengthening the environmental ability to change physical activity‐related cultural norms (Dhair & Abed, 2020).

Females who decide to maintain sedentary lifestyles throughout their reproductive years rather than engaging in regular physical activity, whether light or vigorous, exhibit altered hormonal patterns that have an impact on their physical health. Such hormonal alterations are directly connected to the number of ovarian reserve hormones, which are adversely impacted by inactivity. Age and a lack of exercise can cause the ovarian hormone pool to diminish. AMH appears to be the most significant and useful indication of ovarian reserve among the wide range of biochemical markers. Females who were inactive and performed light aerobic activity did not exhibit a significant change in their E2, LH, FSH, and AMH levels, even after a longer length of time. Heavy activity, on the other hand, lowers AMH levels while raising FSH levels. These findings imply that intense exercise may harm fertility, particularly in females with limited ovarian reserves (Zubair et al., 2021).

#### 2.2.2 Smoking

Smoking is another causative agent that still has controversial backgrounds. There is limited evidence to link decreased fecundity to active male smoking and passive smoking in either spouse. Female smokers who actively smoked, mainly those who had been smoking 10 cigarettes a day for 10 years, had lower fecundity than nonsmokers. While female smoking has been linked to infertility, nothing is known about the dosage and time frame at which an impact is seen. This study evaluated data from a preconception cohort study conducted in North America online that included 5473 female and 1411 male pregnancy planners between 2013 and 2018. At study enrollment, participants had been trying to become pregnant for about six menstrual cycles. On the baseline surveys, they gathered data on past active and passive smoking. Female bimonthly follow‐up surveys asked about pregnancies. Using proportional probability regression models and adjusting for demographic, behavioral, medical, reproductive, and nutritional factors, we obtained fecundability ratios and 95% CI. Fecundity was shown to be somewhat reduced in females who currently smoke regularly, sometimes, or who formerly smoked. The results were more significant for women who smoked 10 or more cigarettes per day for at least 10 years. Reduced fecundity in men was not correlated significantly with current or previous regular smoking, current passive smoking, or either partner's history of smoking. Reduced fecundity among females was related to in utero smoking of fewer than 10 cigarettes per day (Wesselink et al., 2019).

Another study reported that 44% of women and 61% of the males, respectively, had each used marijuana once. The adjusted probability of pregnancy loss was more than twice as high for 317 women (395 cycles) with a positive serum beta‐human chorionic gonadotropin (b‐hCG) who used marijuana at enrolment compared to those who never smoked pot or had smoked it in the past, who had never smoked marijuana or were current users (508/379 cycles). On limited data, this estimate was made. However, regardless of whether the women smoked marijuana or not, couples in which the male partner smoked marijuana at enrollment had a significantly higher adjusted probability of having a live birth than couples in which the male partner had never smoked marijuana. Male and female former marijuana abusers' treatment results did not significantly vary from those of those who had never smoked marijuana (Nassan et al., 2019).

#### 2.2.3 Depression

Infertile women are more likely to experience psychological issues. The importance of sexual function and sexual‐related quality of life is not considered to be important. Depression in infertile women is an important aspect that requires further attention Women have higher levels of anxiety and general distress both before and throughout treatment when the reason for infertility is linked entirely to females, which is presumably connected to guilt. Through better patient counseling and more targeted psychological support, this study aids the treating physician. In this study, two validated standardized questionnaires, the hospital anxiety and depression scale and the fertility quality of life, were administered to 89 women before their first cycle of infertility therapy and once again following the end of ovarian stimulation for IVF. Before receiving therapy, women's anxiety levels were noticeably higher than after. In patients receiving care for female infertility, we observed noticeably higher levels of anxiety and general discomfort. Depending on the primary reason for infertility, they divided the women into three groups (female infertility, male infertility, or both men and women). Earlier in therapy, our group had higher levels of anxiety, which is likely a result of not understanding what they should do to address their problems. In addition, women are more prone to infertility, and she is more likely to feel guilty, thus making anxiety and overall dissatisfaction more severe both before and after therapy (Massarotti et al., 2019).

Common psychological reactions to infertility and medically assisted reproduction include depressive and anxiety emotions. Because the couple feels that it is their responsibility to reproduce and continue the family line, and they feel inadequate if they are unable to have children, homologous depressive and anxiety emotions may be linked to greater levels of distress. Compared to those in heterogonous depressive and anxiety emotions, individuals in homologous depressive and anxiety emotions experienced greater levels of general infertility stress, infertility‐related sexual worries, and depressive/state–trait anxious symptoms. In homologous depressive and anxiety emotions, gender had no influence, but social and sexual anxieties were linked to depressive/trait anxious symptoms. While infertility stress dimensions had no impact, male gender was related to reduced levels of state anxiety symptoms in heterogonous depressive and anxiety emotions (Pozza et al., 2019).

**FIGURE 2:**
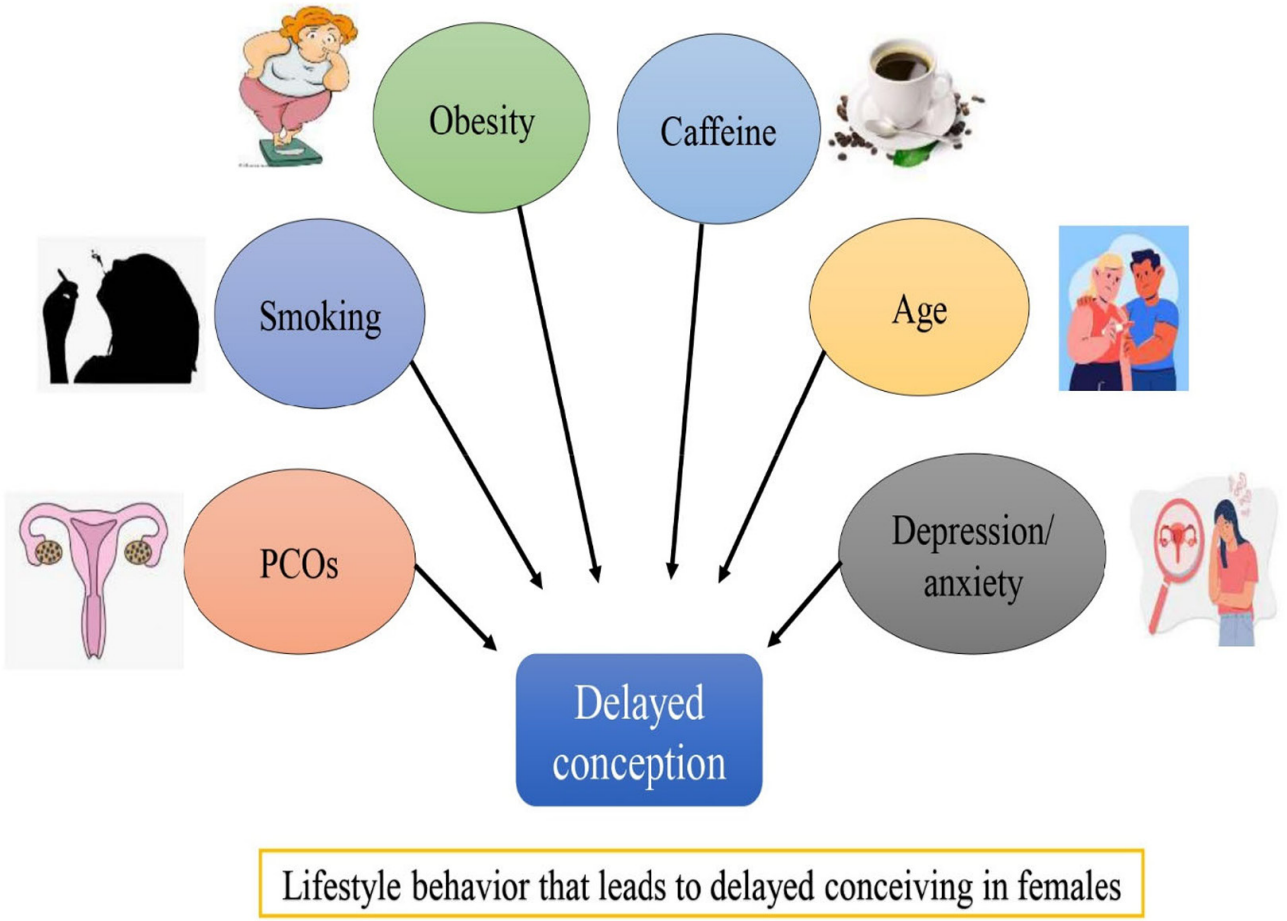
Lifestyle and clinical factors that cause delayed conceiving.

In another study, 240 women from all around Romania who were experiencing fertility issues filled out the STAI‐Form Y, the Brief COPE, and the “Difficulties with Infertility and Its Treatment” scale. According to statistical analyses, individuals who used many fertilization procedures scored lower on the anxiety and difficulty scales than women who were just starting their treatment. It is significant to stress that perceptions of infertility challenges and coping, as well as challenges and state anxiety, are significantly positively correlated. Regarding the connection between state anxiety and coping, adaptive coping techniques had a negative correlation with state anxiety; however, there were strong positive connections between maladaptive coping strategies and state anxiety. Additionally, when it comes to coping mechanisms, women who are aware that female factors play a role in infertility tend to blame themselves and vent. These results highlight the fact that infertile women feel extremely high levels of anxiety and employ a variety of adaptive coping techniques (Iordăchescu et al., 2021). Figure 2 explains the lifestyle and clinical factors that cause delayed conceiving.

#### 2.2.4 Obesity

While talking about the lifestyle and clinical factors behind delayed conception, the first and most dominant one is the duration of obesity as it affects the degree of ovarian dysfunction. Since eating too many meals high in energy may be harmful to one's health over time, obesity is becoming more common worldwide and is associated with early‐age obesity. This study examined the effects of chronic obesity and its consequences on reproductive outcomes. Female Sprague–Dawley rats (aged 21 days) were fed a cafeteria diet (for the obese group) and a standard diet (for the control group) ad libitum for 32 weeks. In maintained obese rats, we found delayed conception, subfertility, macrocosmic pups, subfecundity, and hyperprolactinemia with smaller sizes. The corpus luteum and the number of ovarian follicles (primordial, primary, secondary, and antral follicles) have reduced, which shows that ovulation and folliculogenesis are impaired. Rats that are obese could have less ovarian protein. Their study found that prolonged obesity elevated the severity of reproductive issues by impairing ovulation and folliculogenesis (Kannan & Bhaskaran, 2019).

Moreover, women who are obese and infertile are advised to lose weight before actually trying to conceive and receive infertility treatment to increase the likelihood that they will become pregnant and have healthy babies, although there is a lack of conclusive proof from randomized trials. To have a healthy live delivery, a preconception‐intensive lifestyle intervention with acute weight loss is preferable to a weight‐neutral plan. This randomized control study was conducted between July 2015 and July 2018 on 379 women with obesity (BMI: 30 kg/m^2^) and unexplained infertility. This study was carried out at nine academic health facilities across the United States. The intensive group increased physical activity and lost weight with meal replacements and medication (Orlistat) compared to a control group that increased physical activity but did not lose weight. Among 191 women referred to the normal lifestyle group, 40 dropped out before conceiving; among 188 women assigned to the intensive lifestyle group, 31 dropped out before conception. When compared to standard, intensive resulted in considerable weight loss. Metabolic health improved, with a significant drop in the incidence of metabolic syndrome. In intensive care, gastrointestinal side effects were considerably more prevalent. It is possible that improvements in metabolic health would not result in increased fertility in women (Legro et al., 2022).

In another cross‐sectional study, conducted from April 2015 to March 2017 on 330 infertile women referred to two infertility treatment facilities at Imam Khomeini hospital and Mother Center in Sari, Iran, 54.5% were between the ages of 30 and 39; 55.2% were infertile for 1 to 5 years; 44.5% of the samples had a body mass index (BMI) between 26 and 30 kg/m^2^; and 54.5% had a diploma or an associate degree. Urban dwellers made up 63.6%. Primary infertility was reported by 74.5% of patients. The most common causes and risk factors for infertility were a history of PCOS, pelvic inflammatory disease, and low ovarian reserve, with prevalence rates of 19.42%, 16.81%, and 13.91%, respectively. Thyroid disorders (54.5%) were the most common underlying illness. Residents in urban areas were shown to have a statistically significant association with the length of infertility, endometriosis and educational levels, and miscarriage with thyroid. Chi‐square test (*p* .05) and IBM SPSS 21 software were used for data analysis. In this Iran region, women were obese, uneducated, and had ovarian diseases and thyroid diseases—the main causes of infertility (Marzieh et al., 2021).

#### 2.2.5 PCOS

The next most evident background issue in delayed conception is PCOS. About 20% of infertile couples have females with PCOS health issues. PCOS is one of the primary causes of infertility worldwide. Moreover, first‐trimester spontaneous abortion rates can increase in women with PCOS by 25% to 73% (Al‐mosawi, 2021). PCOS is an endocrine/metabolic condition that is becoming more common. It has several clinical features, the most important of which are androgen excess, oligo‐an ovulatory infertility, polycystic ovaries, insulin resistance (IR), and cardio‐metabolic changes. PCOS is generally associated with obese women, although many PCOS patients are thin. Although each group's metabolic profiles have certain similarities, there are individual variances in each group's body composition and other characteristics (Barrea et al., 2021).

Women with male‐cause infertility, polycystic ovarian syndrome, low AMH levels, and women with unexplained infertility were all included in the research and underwent IVF. The choice of follicular fluid and samples was made on the day of oocyte suction. The amounts of Fe, ferritin, and transferrin as well as the activity of ceruloplasmin ferroxidase were assessed in the follicular fluid. Fe levels in the PCOS group were substantially higher than in the control and UI groups. Ferritin levels in the PCOS group were way greater (*p* .05) than in the control group. Transferrin levels in the PCOS group were substantially higher than in the UI group. Ferroxidase activity showed a lower level but nonsignificant difference when compared to the other groups. In conclusion, women with PCOS may have lower pregnancy success rates while using IVF because of the elevated Fe levels in their follicular fluid (Kadhum & Al‐Shammaree, 2021).

In this study, 264 participants took part in the study (115 healthy controls, 78 with primary, and 71 with secondary infertility). The Beck depression inventory, the female sexual function index, and the sexual quality of life in woman questionnaires were all administered to each participant. In healthy people, the mean Beck depression inventory score was substantially lower. Sexual dysfunction was more common in people with primary infertility, whereas the Beck depression inventory score was substantially higher and the sexual quality of life in females significantly lower. The sexual quality of life in women and overall female sexual function index scores showed a strong positive association. By treating the sexual quality of life in women as dependent and taking age, Beck depression inventory, the length of the marriage, and sexual dysfunction into account, linear regression analysis revealed that Beck depression inventory and sexual dysfunction were independent predictors of the sexual quality of life in women. Consideration should be given to the sexual function and sexual life quality in Iranian infertile couples. More attention should be paid to depression in infertile women as a critical factor (Shahraki et al., 2018).

PCOS is a common syndrome that affects the reproductive age of women worldwide, and it is characterized by metabolic, endocrine, and reproductive problems. It is cross‐sectional research that includes female university students and the respondents were questioned on a few physical indicators and symptoms, including oily skin, weight increase, darker neck skin, excessive hair growth, and acne. Of 100 females, 20% had PCOS but were ignorant of its symptoms; 48% of respondents said they did not have PCOS, although the symptoms they indicated on the questionnaire indicated that they had. When the respondents were questioned about the regularity of their periods, 53.9% reported having irregular periods, 33.3% reported having regular periods but occasionally experiencing irregular periods, and 6.9% had never experienced irregular periods. In contrast to the 15% who were unaware of their weight gain, 45% of individuals reported experiencing weight gain. According to the data, 39.2% of participants experienced an increase in desires, 28.4% of individuals had no cravings, and 30.4% of participants were unaware of their cravings. Cravings are more common in PCOS patients than in normal patients. According to the study's findings, adolescent females typically consume junk food and beverages that interfere with their menstrual cycles and are ignorant of the signs of PCOS (Aslam et al., 2022).

#### 2.2.6 Triclosan and infertility

The percentage of women who met the criteria for assumed infertility was 12.5% overall and 11.7% among all females who were eligible for the research. Urinary triclosan (TCS) levels adjusted for creatinine were considerably greater in individuals who met the infertility criteria than in those who did not. On multivariable adjusted analyses, people with undetectable urinary TCS levels were 35% less likely than people with detectable TCS levels to achieve the required infertility threshold. Comparing the lowest and highest quartiles revealed the largest correlation between TCS levels and infertility. Age‐restricted and weighted analyses maintained the direction and amplitude of the connection between TCS levels and infertility, but the correlations lost statistical significance (Beroukhim et al., 2022).

A common environmental toxin that disrupts the endocrine system is triclosan (TCS). Studies on animals and in vitro showed that triclosan may influence reproduction by interfering with the homeostasis of thyroid and sex hormones. This study was conducted to investigate the relationship between urine triclosan content and the success of in vitro fertilization in women who had visited an infertility clinic. Participants in the research were registered at a Polish infertility clinic. A total of 450 women in the age range of 25 to 45 years gave urine samples. A validated gas chromatography ion–trap mass spectrometry technique was used to assess the urine amounts of triclosan. From patient electronic medical data, clinical results of IVF therapy were extracted. Eighty‐two percent of women had triclosan found in their urine, with a geometric mean of 2.56 6.13 ng/mL. Triclosan levels in the urine were linked to a decline in the implantation rate. Other IVF outcomes including MII oocytes, embryo quality, fertilization rate, and triclosan exposure did not show any correlation (Radwan et al., 2021).

#### 2.2.7 Surgeon females suffering from delayed conception

In another study, this survey included 850 surgeons (692 females and 158 males) in total. Female surgeons with female partners were barred since it was unclear who carried the fetus. Because the included nonchildbearing population was made up of male persons with female spouses, this group is referred to as male surgeons throughout the research. The median (IQR) age was 40 years (36–45). A total of 290 of the 692 female surgeons surveyed suffered a pregnancy loss, which is more than double the incidence of the general population. Female doctors had fewer children than male surgeons were more likely to postpone having children due to surgical training and were more likely to undergo fertility treatment. Female surgeons were more likely to experience serious pregnancy difficulties than female nonsurgeon partners, even after adjusting for age, work hours, in vitro fertilization usage, and multiple gestations. Female surgeons who performed 12 or more hours per week during the pregnancy's third trimester had a greater risk of significant pregnancy problems than those who worked <12 hours per week. Female surgeons were more likely to have musculoskeletal diseases, nonelective cesarean birth, and postpartum depression than female nonsurgeon partners. Infertility and pregnancy‐related medical risks are on the rise among female surgeons, according to nationwide survey research. As more and more women enter the surgical sector, it is critical to change the culture of the field to promote pregnancy to lower the risk of serious pregnancy complications, the need for fertility treatments, and involuntary childlessness as a consequence of delayed attempts at parenting (Rangel et al., 2021; Table 2).

**TABLE 2:** Lifestyle factors that cause delayed conceiving.

Factor	Participant	Methodology	Result	References
Physical activity	20 females (25–35 years)	Divided into two groups, first group doing light aerobic, second group doing heavy exercise	A light exercise and sedentary did not hormonal changes. Heavy exercise decreased the AMH and FSH levels which led to low overran reserve.	Zubair et al. (2021)
Smoking	5473 females and 1411 males (21–45 years)	Internet‐based prospective cohort study	Both active and passive smoking cause fecundability.	Wesselink et al. (2019)
Depression and anxiety	89 women	The HADS and FertiQoL are two verified standardized questionnaires that were used	Women are more likely to feel guilty and have greater levels of anxiety and overall stress both before and after treatment.	(Massarotti et al., 2019)
Depression	264 hospitalized women (18–40 years of age)	Females were divided into healthy control, secondary, or primary infertility. Fill out the form by using the Beck depression inventory, sexual quality of life in females, and female sexual function index score.	Depression influences fertility. More attention should be paid to depression as a critical issue in infertile women	Shahraki et al. (2018)
TCS	Females from UN (measure the level from 2013 to 2016)	TCS mostly uses houses for different purposes that affect the endocrine system of reproduction. The survey was conducted for analysis of TCS levels in the body through analysis of urine, plasma, and breast milk.	There is a strong relationship between TCS and infertile. A high TCS levels of females conceive cannot be over 1 year.	Beroukhim et al. (2022)
TCS	Females in age 25–45 years old	450 females were involved in this research. Their urine samples were taken by using the gas chromatography ion–trap mass spectrometry technique	82% of females had TCS in their urine. TCS levels reduced the implantation rate, while not showing any relation with IVF treatments	Radwan et al. (2021)
Obesity	Sprague–Dawley female rats (21 days old) divided into two groups, first (control) group and second (obese) group	A randomized control study was conducted for 32 weeks in female rats. Were fed a regular diet (control group) or cafeteria diet (obese group) ad libitum	Obesity reduces ovulation and folliculogenesis Raising the severity of reproductive impairments	Kannan & Bhaskaran (2019)
Obesity	379 obese women (16 days trials) In two treatment groups	A randomized control study was conducted on obese females; they were divided into two different groups Lifestyle interventions in one group through increased physical activity and medications	Weight loss did not enhance fertility or delivery outcomes	Legro et al. (2022)
Females who were not conceived after 18 months of the follow‐up period	1530 women	A survey‐based study by using questionnaire data was collected and then analyzed them. That also addresses the Sustainable Development Goals.	This helps to better understand the attributes, experiences, and parenting techniques of the study community's women who have delayed conception.	Adhikary et al. (2022)
The delayed attempt of conceiving due to carrier	850 surgeons (692 women and 158 men)	From November 2020 to January 2021, this survey questionnaire was electronically circulated and gathered among males and females attending. And resident surgeons with children through different surgical societies in the United States and social media	Female surgeons faced with higher medical risks of infertility and pregnancy difficulties. A reduction in the chance of severe pregnancy complications and involuntary childlessness due to postponed attempts at childbearing	Rangel et al. (2021)

## 3 CONCLUSIONS

The review explaine about the increasing problem for practically all young ladies globally is delayed conception. Delay in conception is caused by a variety of variables, including nutritional, lifestyle, and medical issues. Dietary factors such as deficiencies in vitamins and minerals contribute to problems in conceiving. While many studies indicated that if females take folic acid or Fe supplements, it is useful to reverse their conditions, it may be reversed by taking supplements and eating a balanced diet that is high in these essential vitamins and minerals. Vitamin D deficiency in females who do not get enough sun also relates to delayed conception. The issue is that obese persons on a low‐zinc diet may be more at risk for infertility. When discussing lifestyle issues, it is important to note that a lack of physical exercise or an excessively active lifestyle might affect fertility. Therefore, walking and moderate exercise are beneficial for preventing obesity and living an active lifestyle. Another causative factor that continues to be debated is smoking. Less than 10 cigarettes a day of in utero smoking was linked to decreased fertility in females. A significant contributing factor to delayed conception in females is the medical condition. In medical, diabetes, hypertension, PCOS, and other diseases and their medicine for prevention seem to be the main factors that contribute to delayed conception in females. Infertility is mostly caused by inadequate nutrition and a bad lifestyle among our young people, both of which influence ovarian reserves. To prevent delayed conception, eat a nutritious and balanced diet rich in key nutrients, engage in physical activity, and avoid smoking, PCOS, and medical issues.

## AUTHOR CONTRIBUTIONS

**Maryam Khalid Rizvi:** Conceptualization (equal); formal analysis (equal); resources (equal); software (equal); validation (equal); visualization (equal); writing – original draft (equal); writing – review and editing (equal). **Roshina Rabail:** Conceptualization (equal); data curation (equal); investigation (equal); methodology (equal); visualization (equal); writing – original draft (equal); writing – review and editing (equal). **Seemal Munir:** Validation (equal); visualization (equal); writing – original draft (equal). **Muhammad Waleed Rizvi:** Data curation (equal); formal analysis (equal); funding acquisition (equal); resources (equal); software (equal); visualization (equal); writing – original draft (equal). **Rana Muhammad Aadil:** Conceptualization (equal); data curation (equal); formal analysis (equal); supervision (equal); validation (equal); visualization (equal); writing – original draft (equal). **Gholamreza Abdi:** Conceptualization (equal); data curation (equal); formal analysis (equal); funding acquisition (equal); methodology (equal); supervision (equal); validation (equal); visualization (equal); writing – original draft (equal); writing – review and editing (equal).

## CONFLICT OF INTEREST STATEMENT

None.

## Data Availability

Not applicable.
